# Community places visited and abandoned by people living with dementia in British Columbia, Canada: patterns and implications

**DOI:** 10.3389/fpubh.2026.1863001

**Published:** 2026-07-23

**Authors:** Habib Chaudhury, Tianci Dong, Shawna Hopper, Joey Wong, Kishore Seetharaman, Cari Randa-Beaulieu, Emma Rossnagel, Lillian Hung, Mark Groulx, Ji Young Cho, Shannon Freeman

**Affiliations:** 1 Department of Gerontology, Simon Fraser University, Burnaby, BC, Canada; 2 Department of Housing and Interior Design, Kyung Hee University, Dongdaemun, Republic of Korea; 3 School of Nursing, University of British Columbia, Vancouver, BC, Canada; 4 Alzheimer Society of British Columbia, Vancouver, BC, Canada; 5 School of Nursing, University of Northern British Columbia, Prince George, BC, Canada; 6 School of Planning and Sustainability, University of Northern British Columbia, Prince George, BC, Canada

**Keywords:** act-out, aging, Canada, community participation, dementia, out-of-home activities and places

## Abstract

**Purpose:**

This study explores patterns of community participation and abandonment among older adults living with mild to moderate dementia in British Columbia, Canada. It identifies types of out-of-home places that are retained and/or abandoned over time, as well as the reasons for ongoing participation or the abandonment of places.

**Methods:**

The study uses the ACT-OUT survey and the participants included 32 community-dwelling individuals living with dementia. All participants described the places they visited across four areas—consumer, medical, social, and recreational—reflecting on their past, current, and expected future participation. Patterns in the data were examined using one-sample t-tests.

**Results:**

Significant declines in participation were observed from past to present in most categories, particularly for administrative offices (e.g., government buildings, city halls, and other bureaucratic service centers), sports facilities, places of worship, and hospitals. Commonly retained places included neighborhoods, parks, senior centers, and doctor’s offices, largely due to familiarity, proximity, and necessity. Anticipated future participation showed stability or modest improvement in similar categories.

**Conclusion:**

The findings highlight a narrowing of community engagement for people living with dementia, which emphasizes the importance of dementia-friendly community initiatives that prioritize familiar, accessible, and supportive environments to sustain meaningful out-of-home participation.

## Introduction

Globally 57 million people live with dementia and around 10 million new cases of dementia are reported each year (1). In Canada, approximately 771,939 individuals are living with dementia, and the number is expected to increase to 1 million by 2030 (2). Provincially, similar trends can be seen in British Columbia (B. C.), Canada, where the estimated number of cases will increase from 85,800 in 2022 to 247,300 by 2050 (3). The increased prevalence of persons living with dementia around the world emphasizes the urgency to create supportive and caring communities that can foster maintenance of independence and social engagement as long as possible and delay potential need for institutional relocation.

Dementia-friendly communities (DFCs) aim to reduce stigma, improve accessibility and mitigate challenges related to living with cognitive impairment. Dementia-friendly environments can be created through supportive infrastructures (e.g., safe and accessible sidewalks, benches, landmarks for spatial orientation), national policies (e.g., early screening, caregiver education), and inclusive services and programs (e.g., community-based programs, services, activities) (4, 5). Initiatives like Alzheimer’s Cafés and memory-friendly walking groups have shown success in promoting social engagement and well-being of older adults living with dementia (6, 7). However, maintaining out-of-home participation and activities can be challenging for people living with dementia, due to decline in cognitive functioning over time, built environmental barriers, and underlying societal stigma (8). Social participation is important for maintaining independence and reducing the level of social isolation; however, existing assessment tools prioritize in-home activities or dementia caregiver proxies, neglecting community involvement (9–11).

The “Participation in Activities and Places Outside Home for Older Adults” (ACT-OUT) survey addresses this gap by evaluating out-of-home engagement across four domains of places, including: (A) consumer, administrative, and self-care places; (B) places for healthcare; (C) social, spiritual, and cultural places; and (D) places for recreational and physical activities. The full list of the destinations is provided in Table 1 (12, 13).

**Table 1:** Percent difference in participation in places for past (B), present (P) and future (F).

Activity/Place	Before (%)	Present (%)	Future (%)	B–P Δ% (*p*-value)	P–F Δ% (*p*-value)
A. Consumer, administrative, and self-care places					
Small grocery store (A1)	88	63	69	−25 (0.007)	+6 (NS)
Mall supermarket, big store (A2)	97	78	78	−19 (0.017)	0 (NS)
Small store (A3)	94	75	72	−19 (0.022)	−3 (NS)
Pharmacy (A4)	100	78	81	−22 (<0.001)	+3 (NS)
Hairdresser salon (A5)	94	78	84	−16 (0.044)	+6 (NS)
Bank/post office (A6)	100	78	75	−22 (<0.001)	−3 (NS)
Administration places (A7)	75	25	41	−50 (<0.001)	+16 (NS)
B. Medical care places					
Doctor office (B1)	97	90	97	−7 (NS)	+7 (NS)
Hospital/healthcare centre (B2)	94	59	75	−35 (<0.001)	+16 (NS)
Dentist’s office (B3)	97	84	88	−13 (NS)	+4 (NS)
Therapy (B4)	75	28	50	−47 (<0.001)	+22 (0.011)
Day Care (B5)	50	38	59	−12 (NS)	+21 (0.017)
C. Social, cultural, and spiritual places					
Friend/family members place (C1)	84	72	81	−12 (NS)	+9 (NS)
Restaurant, Café/bar (C2)	97	91	97	−6 (NS)	+6 (NS)
Senior centre, social club’s premises (C3)	75	67	81	−8 (NS)	+14 (NS)
Building for worship (C4)	72	28	38	−44 (<0.001)	+10 (NS)
Cemetery, memorial place (C5)	47	25	41	−22 (0.008)	+16 (NS)
Entertainment/cultural place (C6)	97	75	84	−22 (0.008)	+9 (NS)
D. Recreational and physical activities places					
A garden in your backyard (D1)	72	50	50	−22 (0.021)	0 (NS)
A park, green areas, community garden (D2)	88	84	84	−4 (NS)	0 (NS)
A forest, mountain, lake, seaside (D3)	84	59	75	−25 (0.008)	+16 (NS)
A cottage, summerhouse, chalet/allotment (D4)	41	28	31	−13 (NS)	+3 (NS)
A neighborhood (D5)	88	88	84	0 (NS)	−4 (NS)
A sport facility (D6)	84	44	50	−40 (<0.001)	+6 (NS)
A transportation centre (D7)	91	66	69	−25 (0.006)	+3 (NS)

B–P Δ% = Percentage point change from Before to Present; P–F Δ% = Percentage point change from Present to Future; NS = Not Significant.

The tool is grounded in the ecological model of aging (14) and applies the person-environment interaction principle to design and advocate for neighborhoods that support well-being for people living with dementia. It uses a validated self-report survey uniquely designed to capture the dynamic interplay among individuals, activities, and their surrounding environments. It has been used for cognitive interviews with older adults both living with and without dementia to ensure content validity (15). The tool has been validated in cross-cultural settings including Switzerland, Sweden, the United Kingdom and Canada, which has established its utility, and shown a tendency for people living with dementia to abandon recreational and medical venues due to sensory challenges and loss of familiarity as dementia processes with time (15, 16). Additionally, the tool’s utility in detecting differences in participation, place abandonment, and perceived risks between people living with and without dementia demonstrated its discriminant validity (15, 17). Collectively, these findings indicate ACT-OUT is a valid and reliable instrument for assessing complex dimensions of out-of-home participation in older adults, especially for older adults with cognitive impairments.

This study presents findings based on the ACT-OUT survey, conducted as part of a larger project “(blinded for review),” which explored the walking experiences of people living with dementia in their neighborhoods (blinded for review). In contrast to prior tools, such as Community Integration Questionnaire (CIQ) and Community Participation Indicators (CPI) (18–20), ACT-OUT emphasizes self-reported experience from community dwelling older adults living with dementia, which enables people living with mild-to-moderate dementia to articulate barriers they have encountered in out-of-home engagements. Previous studies have been limited to larger-scale or caregiver-reported data in a relatively shorter timeframe (15, 21).

The study objectives were to: (i) examine patterns of out-of-home participation among older adults with dementia across four domains with a range of place types (e.g., small grocery stores, doctor’s office, and community garden settings etc.), (ii) compare past, present, and predicted future participation trends in various place categories to understand how engagement and disengagement changes over time for older adults living with dementia, and (iii) identify which types of places are most frequently retained or abandoned as individuals experience cognitive decline, and the reasons behind these shifts (e.g., distance, familiarity, physical barriers, and/or social isolation).

## Materials and methods

Participants were recruited as part of a mixed-methods study designed to examine mobility in the neighborhood and community participation among older adults living with dementia in British Columbia (BC), Canada. A total of 32 participants were enrolled, including 26 from Metro Vancouver, BC and six from Prince George, BC. This paper reports on data collected from people living with dementia and feedback from participant-care partners (dyads). To ensure optimal recruitment, the recruitment and sampling did not discriminate based on the various types of dementia diagnosis (blinded for review).

The study received harmonized ethics approval from the institutional research ethics boards at [university name blinded for review]. The study adopted a process consent approach where informed consent (i.e., on-going written and/or verbal) was obtained from participants and/or their care partners at each stage throughout the research process (22–24, 54). For participants living with dementia who were unable to provide written consent, verbal assent was obtained and audio recorded. The verbal assent was also supplemented with written informed consent from their care partners (22, 25). During the data collection process, participants could choose to have their care partners take part for direct or indirect support. All participation was voluntary, and participants were able withdraw at any time without consequence.

### Inclusion criteria, recruitment, and interview considerations

The study used purposive sampling to ensure the inclusion of participants who could provide “appropriate and useful information” relevant to the research objectives (26) based on the following inclusion criteria: (i) a self-reported diagnosis of mild to moderate dementia or mild cognitive impairment, (ii) current residence in Metro Vancouver community or Prince George BC, Canada, and (iii) regular or occasional practice of walking outdoors. The team adopted various recruitment strategies, including the distribution of promotional leaflets to older adult dementia support groups, programs at local senior centers and dementia advocacy organizations. Detailed information about the study was presented at dementia support group meetings (27). In addition, participants were recruited through the University of British Columbia Hospital Centre for Alzheimer Disease and Related Disorders (UBCH CARD), where clinicians identified eligible individuals who were subsequently contacted by the research team. Participants who learned about the study through community outreach could contact the research team via email or telephone using the contact information provided in the flyer or presentation (28).

All respondents were screened by the research team over the phone to ensure they met the inclusion criteria. Interviews were conducted in-person or virtually to explore participants’ engagement with their neighborhood environment, and the structured ACT-OUT questionnaire was closely followed through the interview process (15). The in-person interviews took place at the research institution or at a location preferred by the participant (e.g., their home, a local café, or the researchers’ institution); the virtual interviews were conducted through the Zoom web conferencing platform. Two-three trained research assistants (RA) conducted the interviews with the study participants at a location of their choice (e.g., their homes, local libraries, community centres, cafés). The RAs had experience in working with people living with dementia and adopted effective communication strategies to enhance the quality of response. The interview strategies included: (i) ensuring a private and comfortable interview process; (ii) taking time to build rapport and pacing the interview to match the participant’s cognitive capacity; (iii) slowing down and rephrasing questions when needed; and (iv) paying close attention to signs of confusion, word-finding difficulty, or anxiety. Interviews were carried out with empathy and patience to maximize participant comfort and ongoing engagement (29). At the beginning of each interview session, all participants were informed that they could withdraw from the interview at any time.

### Outcome measures

This study utilized the ACT-OUT survey tool to assess place-based participation for community-dwelling individuals with mild to moderate dementia in Metro Vancouver and Prince George. The questionnaire is structured into three parts: (i) Part one consists of various types of neighborhood places where participants visit for meaningful activities, (ii) Part two includes two sets of parallel surveys after participants’ dementia diagnosis to determine (a) the places where they report no change in frequency of visits, and (b) the places where they have experienced or anticipate a reduction in visits (15); the data on participation trajectories were collected across past, present, and anticipated future timepoints; (iii) Part three collects data on a broader scope regarding participants’ attitudes on perceived risk, self-rated life satisfaction, and any concerns about staying out in public spaces (15, 17). Self-rated health was assessed using a 5-point Likert-type scale with 1 being “Excellent” and 5 being “Poor” (30). The survey took approximately 25–30 min. ACT-OUT has been proven as a robust and sensitive tool to measure dementia-related mobility and participation with an emphasis on the time trajectories of activity (15, 17).

### Data analysis

Quantitative analyses were performed using R version 4.2.2 (31). One-sample t-tests were conducted to determine statistically significant changes in participation in each place over time (past to present, present to future). Results where *p* < 0.05 (*p*-value threshold) were considered statistically significant. Additionally, net participation differences (calculated by subtracting present participation counts from past participation counts) were used to identify destinations that were abandoned or retained. Given the exploratory nature of this study and the modest sample size, we did not apply corrections for multiple comparisons (e.g., Bonferroni) to avoid an increased risk of Type II errors.

## Results

The study sample included 15 females (47%) and 17 males (53%) for a total of 32 participants, with a mean age of 77 years and a standard deviation (SD) of 8 years (See Table 2 for descriptives). Fifteen care partners among the 32 participants also participated in a dyad setting during the survey session. The majority of participants self-identified as White (75%). Other backgrounds reported included East Asian (16%), Southeast Asian (3.1%), Middle Eastern (3.1%), and mixed-race (3.1%). Most participants reported being married (66%) and retired (88%). Educational backgrounds were very diverse: approximately 37% of participants graduated from post-secondary and 25% reported having a college diploma. Regarding annual income, 26% of the sample reported an annual earning over $70,000 and 23% reported earning between $40,000 and $49,999. Functional health limitations were common among the participants. 72% of individuals reported health conditions that restricted their ability to perform activities of daily living (ADLs), compared to 28% who reported no limitations. Participants rated their current health as: excellent (19%), very good (22%), good (28%), fair (28%), and poor (3.1%). These assessments aim to provide insights on perceived well-being in relation to their functional and socioeconomic engagements (32).

**Table 2:** Participant characteristics (*N* = 32).

Variable	Mean (SD); *n* (%)
Age	77 (8)
Gender	
Female	15 (47%)
Male	17 (53%)
Race	
White	24 (75%)
East Asian	5 (16%)
Southeast Asian	1 (3.1%)
Middle Eastern	1 (3.1%)
Mixed Race	1 (3.1%)
Relationship status	
Never married/Single	2 (6.2%)
Married	21 (66%)
Cohabitating	3 (9.4%)
Separated/Divorced	5 (16%)
Widowed	1 (3.1%)
Education	
PhD	1 (3.1%)
Masters	1 (3.1%)
Undergraduate	10 (31%)
College Diploma	8 (25%)
Some College	4 (12%)
High School Graduation	6 (19%)
Some High School	2 (6.2%)
Employment status	
Unemployed and not looking for work	2 (6.2%)
Retired	28 (88%)
Disabled, not able to work	1 (3.1%)
Self Employed	1 (3.1%)
Household income	
$10,000 to $14,999	1 (3.2%)
$15,000 to $19,999	1 (3.2%)
$30,000 to $39,999	2 (6.5%)
$40,000 to $49,999	7 (23%)
$50,000 to $59,999	5 (16%)
$60,000 to $69,999	1 (3.2%)
$70,000 and over	8 (26%)
Prefer not to say	6 (19%)
(Missing)	1
Health conditions limiting ADLs	
Yes	23 (72%)
No	9 (28%)
Self-rated general health	
Excellent	6 (19%)
Very Good	7 (22%)
Good	9 (28%)
Fair	9 (28%)
Poor	1 (3.1%)

The findings include various destinations visited by participants, categorized into four domains: (A) consumer, administrative, and self-care places, (B) medical care places, (C) social, cultural, and spiritual places, and (D) recreational and physical activity places. Descriptive statistics for participation in each place in these four domains across the three time points (i.e., past, present, and future) over the trajectory of cognitive decline are summarized in Table 1.

### A. Consumer, administrative, and self-care places

Participants actively attended six out of the seven places listed in this domain, with the lowest present participation at administration places. In this study, “administration places” refer to locations, such as government buildings, city halls, social security offices, and other bureaucratic service centers where people go to handle legal documents, permits, or administrative tasks. The largest significant decrease of 50% (75% in the past and 25% at present; *p* < 0.001) was also seen at administration places. Statistically significant differences were noted between past and present participation among all places in the category: participation in small grocery stores dropped from 88% before diagnosis to 63% at present (*p* = 0.007); similar trends were observed for mall supermarkets (97 to 78%, *p* = 0.017), small stores (94 to 75%, *p* = 0.022), pharmacies (100 to 78%, *p* < 0.001), hair salons (94 to 78%, *p* = 0.044), and banks/post offices (100 to 78%, *p* < 0.001). Several reasons for the decline explained by participants include cognitively challenging tasks, such as navigating in environments perceived as over stimulating, and lack of dementia-friendly signage at destinations.

The levels of participation at all seven places were not predicted to significantly change in the future. Frequencies of future participation were predicted to have a minor decrease of 3% in both small stores and bank/post offices. The reasons included limited staff support and stigma associated with both places. However, for a few places, such as the hairdresser salon, participation was predicted to increase slightly (less than 10%) in the future as it was noted by participants that despite the cognitive decline, they were still willing to develop coping strategies that would allow them to selectively engage in familiar activities and maintain a sense of dignity.

### B. Medical care places

Present attendance was highest at the doctor’s office (90%) and dentist (84%). Many participants reported long-term trusting relationships with their healthcare providers due to familiarity built over time. Significant declines were noted for hospitals or healthcare centres (94 to 59%, *p* < 0.001) and therapy services, which include physical, occupational, or speech therapy, and other rehabilitative services (75 to 28%, *p* < 0.001). The main reasons mentioned by participants were that they rely on their primary care providers due to the cognitive decline, while others cited that the transportation and logistics barriers hinder their visits.

Participation at all places in this domain were predicted to increase between present and future, with statistically significant differences reported for therapy and day care. “Day care” refers to adult day programs or centers that provide structured social and health-related activities during the day for older adults, while also offering support and respite for family caregivers. Notably, despite a decline in therapy use between past and present, it was anticipated to show a statistically significant rebound in the future (50%, *p* = 0.011). Some participants cited that therapy could help them maintain independence for a longer period of time despite their cognitive decline, while other participants noted that they might reconsider going to therapy if they could receive assistance or it was strongly recommended by their physician. The expected further increase in this domain may reflect the participants’ optimism, perceived independence, and anticipated external support.

### C. Social, cultural, and spiritual places

Visiting restaurants, senior centres, and cafés, as well as social visits to friends and family, remained stable with no significant changes reported. However, statistically significant declines were found for participation in buildings for worship (72 to 28%, *p* < 0.001) and cemeteries or memorial sites (47 to 25%, *p* = 0.008). Participants may not have visited these locations due to accessibility issues, as the destinations were outside their neighborhoods and required traveling to other parts of the city. There was also a significant decrease in visiting entertainment/cultural places (97 to 75%, *p* = 0.008). The decrease in attendance at entertainment or cultural places for many participants was due to the distance and accessibility: participants mentioned that they tried to avoid larger crowds and had a hard time navigating buildings without elevators or escalators.

The levels of participation at all six places in this domain were not predicted to significantly increase in the future. This is mainly because participants anticipate a continued disengagement and reduction in social circles as their cognitive functions decline. Some participants also stated that they felt these activities were no longer meaningful or necessary to prioritize.

### D. Recreational and physical activity places

Participation at recreational and physical activity places varied. High participation was reported at parks, green areas, and community gardens (84%), as well as within the neighborhood (88%) at present. The net difference in the use of parks, community gardens, and neighborhood walking areas remained relatively stable from the past to the present. Many participants cited that these places were relatively familiar, accessible, and less stressful, and enabled them to follow an established routine. However, use of backyard gardens dropped from 72 to 50% (*p* = 0.021), and visits to forests, lakes, or other natural spaces declined from 84 to 59% (*p* = 0.008). The reason as stated by participants was due to household downsizing (e.g., from a house to an apartment) and reduced physical mobility. Use of sport facilities showed one of the most significant drops from 84 to 44% (*p* < 0.001). These environments, such as gyms and pools were viewed as fast-paced and stress-inducing; participants and their care-partners expressed fear of injury, especially at places where staff may lack dementia-awareness.

The levels of participation at all seven recreational places were predicted to have no significant increase in the future. Several participants indicated that the current routines already reflected their comfort zone, while other participants and/or participant-care partners (dyads) even expressed thoughts of shrinking the scale of participation further given the progressive physical and cognitive decline.

### Evaluation of places abandoned or retained

Table 3 summarizes the net differences and rates of participation in the past and present for each place within the four domains. The results are reported across three time points, including the past, present, and future. The past/present net participation indicates that all destinations were abandoned by participants residing in the same neighborhood. Participants were most likely to abandon administration places (−16), therapy (−15), and buildings for worship (−14), and sport facilities (−13). Several consumer and recreational locations (small grocery stores, banks, pharmacies, and natural outdoor areas) also showed moderate declines with an average loss of six to eight participants in each place. In contrast, the places most frequently retained i.e., with little or no change in participation included participants’ neighborhoods (0), parks and community gardens (−1), senior centres (−2), restaurants and cafés (−2), and doctor’s offices (−2). The results suggest greater continuity and engagement in activities associated with daily routines and familiarity.

**Table 3:** Evaluation of destinations abandoned or retained.

Place–domain	Past/Present (Net Figure)	Places abandoned
Administration places - A	24/8 (−16)	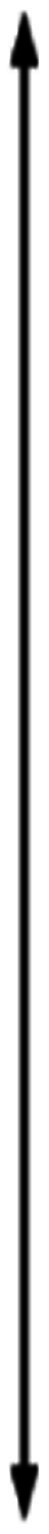
Therapy - B	24/9 (−15)	
Building for worship - C	23/9 (−14)	
A sport facility - D	27/14 (−13)	
Hospital/healthcare centre - B	30/19 (−11)	
Small grocery store - A	28/20 (−8)	
Bank/post office - A	32/24 (−8)	
A forest, mountain, lake, seaside - D	27/19 (−8)	
A transportation centre - D	29/21 (−8)	
Pharmacy - A	32/25 (−7)	
Cemetery, memorial place - C	15/8 (−7)	
Entertainment/cultural place - C	31/24 (−7)	
A garden in your backyard - D	23/16 (−7)	
Mall supermarket, big store - A	31/25 (−6)	
Small store - A	30/24 (−6)	
Hairdresser salon - A	30/25 (−5)	
Dentist’s office - B	31/27 (−4)	
Day Care - B	16/12 (−4)	
Friend/family members place - C	27/23 (−4)	
A cottage, summerhouse, chalet/allotment - D	13/9 (−4)	
Doctor office - B	31/29 (−2)	
Restaurant, Café/bar - C	31/29 (−2)	
Senior centre, social club’s premises - C	24/22 (−2)	
A park, green areas, community garden - D	28/27 (−1)	
A neighborhood - D	28/28 (0)	
		Places retained

## Discussion

This study examined changes in participation across a wide range of out-of-home destinations among older adults with mild to moderate dementia in British Columbia, Canada, using the ACT-OUT tool to compare past, present, and anticipated future engagement. Consistent with previous findings, there was a pattern of decline in participation from the past to the present across all four domains: (A) consumer, administrative and self-care places, (B) medical care places; (C) social, cultural, and spiritual places; and (D) recreational and physical activity places (10, 17). Anticipated future participation was slightly reduced, stable and/or with modest recovery in a few locations, suggesting that many of these losses in engagement may become long-term or permanent without intervention (17, 33).

Statistically significant declines in past-to-present participation were observed across several places in the four domains, including (i) visits to pharmacies, banks, and administrative offices in domain A (consumer, administrative and self-care places), (ii) visits to hospital and therapy services in domain B (medical care places), (iii) participations in places of worships, cemetery, or entertainments in domain C (social, cultural, and spiritual places), and (iv) visits to sport facilities in domain D (recreational and physical activity places). In relation to places of abandonment and retention, the results are aligned with the previous ACT-OUT studies, which found that people living with dementia tend to withdraw from community destinations across all domains over time (17, 34). In terms of place abandonment and retention, the findings from this study support existing evidence that participants with dementia are more likely to discontinue visiting places, especially to the ones requiring high cognitive effort (i.e., shopping and service centres) and physical strength (i.e., gym or fitness centres), followed by medical care facilities (i.e., hospitals and therapy services) and social or consumer locations (10, 35). There could be several factors contributing to cognitive challenges in these places, based on particular tasks, interactions and/or activities in each of these places. For example, a person living with dementia may face multiple cognitive demands in a grocery store, such as wayfinding to locations of desired products, choosing a particular product from a variety of options, paying the cashier with the right amount of currency or using the bank card. Hence, the social interactions, physical environmental features, product options and placement are all part of the grocery shopping experience that can be challenging and potentially overwhelming for a person living with dementia at some stage in their cognitive journey. Likewise, there would be another set of social aspects and socio-physical environmental demands in another place type (e.g., pharmacy or doctor’s office) that would likely contribute to cognitive challenges leading to anxiety and frustration. Such challenges of functioning in social spaces are congruent with associated with loss of functional reserve and diminution of functional status (36).

Participants living with dementia faced significant challenges in various locations, which resulted in abandoning these places across all four domains in the study. The places with the high level of abandonment risk included administrative offices, places of worship, and sports facilities. Participants mentioned that environmental and sensory barriers, such as noise, lack of signage and obstruction on roads, can also make outdoor participation more difficult as dementia progresses and cognitive function deteriorates. Additionally, en-route challenges were noted, such as the fear of being lost and disorientation. Navigating unfamiliar or complicated environments, such as public transportation, crossing busy streets, or managing unfamiliar environments, can create anxiety and confusion for individuals living with dementia.

The places most commonly retained were neighborhoods near participant’s residence and doctor’s offices (often with the support of care partners) due to the accessibility, familiarity and necessity. The continued use of green spaces and senior centers also reflects a preference for nature-based and low-stress environments that support social interaction. Supporting access to these retained places while addressing accessibility transportation and dementia-friendly city design could promote greater community participation. Collectively, statistically significant decreases were primarily observed between the past and present participation, while anticipated future participation levels showed either stability or modest improvement in some categories. This pattern suggests that after an initial period of adaptation, older adults living with dementia may establish new routines and develop coping strategies that allow them to maintain a stable level of participation in certain places. These findings show a consistent pattern of reduced community involvement in more formal, structured, and/or cognitively demanding environments for the participants in the context of past and present. The stability or modest improvement indicates a sense of optimism of continuity with the current patterns of community engagement.

The patterns identified in this study contribute to our understanding that community environments become increasingly narrowed for people with dementia as their condition with dementia progresses over time (17, 37). This shows a consistency with previous findings, which indicate that people living with dementia experience reduced mobility in the community and social inclusion over time (17, 33, 38). The findings reaffirm the need for proactive dementia-friendly community (DFC) interventions and better environmental designs to prevent social isolation and disengagement of older adults living with dementia. Furthermore, this study demonstrates the utility of the ACT-OUT tool in identifying high-priority locations to sustain community participation over long term trajectories (17).

This study identified specific factors that facilitated continued participation in different out-of-home destinations among participants living with dementia. First, familiarity with the environment and long-term trusting relationships with service providers, such as doctors and dentists was consistent with prior research, suggesting that familiar and routine settings can reduce confusion, improve comfortability, and manage anxiety levels for individuals living with dementia (8, 39). Second, destinations located closely to participants’ homes, such as local parks, neighborhood sidewalks, and nearby cafés, were more frequently retained: the evidence suggested that physical accessibility and walkability were essential for maintaining autonomy and social inclusion (33, 40). Continued use of such places demonstrates the importance of designing dementia-inclusive environments that prioritize familiarity, accessibility, and supportive social interaction, which is the key to sustain community engagement for older adults living with dementia over time. Overall, these findings reinforce the importance of designing dementia-friendly communities that actively reduce environmental, social, and structural barriers, as well as facilitate daily community participation. It is important to ensure that destinations remain accessible through initiatives, including improved dementia-friendly signage, accessible transport, and staff training to help prevent premature disengagement (17, 33). As discussed above, the results were consistent with prior research findings that familiarity, routine procedures, and positive past experiences acted as key facilitators of continued community participation (40).

### Limitations

While participants were encouraged to complete the ACT-OUT survey independently, a few interviews needed to include a caregiver, whose presence, while supportive, occasionally changed the responses. In a few cases, participants looked to caregivers for help or approval when they were unable to remember. To address this in the analysis, we noted instances where caregivers contributed to the responses; however, a systematic comparison of independently completed versus caregiver-assisted surveys was not feasible due to the sample size. Future studies with larger samples could explore this further. F Although self-report data collection provides insights of the lived experience, it can introduce potential recall bias and inaccuracies, particularly in retrospective accounts of past activities. Additionally, survivorship bias likely skewed results toward healthier and more engaged community-dwelling individuals, as those who face more health conditions and severe environmental barriers would be less likely to make a trip to participate in the survey. Finally, the use of *p*-values without corrections for multiple comparisons may increase the risk of Type I errors (false positives).

## Conclusion

The ability to engage in meaningful out-of-home activities is critical to the health, independence and quality of life for people living with dementia (41). This study highlights the increasing trend of community disengagement across various domains and points out the importance of targeted interventions and dementia-inclusive planning. Given the limited empirical research on out-of-home participation among people living with dementia in Canada, this study contributes to our evolving understanding of the types of destinations people living with dementia engage and do not engage with, shifts in participation over time, and factors (both facilitators and barriers) influencing these shifts. The results from this study can be used to help better support community participation for older adults living with dementia by identifying specific destinations at risk of abandonment. Further, the results provide insights for policy makers, health practitioners, social workers, and family caregivers as to which community destinations are beneficial for individuals with dementia, and which are at higher risks of abandonment and thus require targeted interventions for supportive social and physical environments.

The study highlighted the importance of proximity, familiarity, and supportive design in maintaining community participation for older adults living with dementia. It reinforced the need for dementia-inclusive environments at municipal/city levels, including having more dementia-inclusive signage, accessible transportation, and staff support. While these findings offer a useful starting point, future research is needed to explore how community-dwelling individuals with dementia can better navigate their environments and adapt to challenges as dementia progresses. Facilitating outdoor participation is not only crucial for enhancing their quality of life, but also necessary to promote inclusion and awareness of dementia to the public. Supporting individuals to maintain their mobility as active citizens within communities is a key component of the DFC movement and an important step to reducing stigma (42) - a goal to ensure that people living with dementia remain seen, valued, and connected in the world.

## Data Availability

The raw data supporting the conclusions of this article will be made available by the authors, without undue reservation.
